# Characterization of the Dynamic Transcriptome of a Herpesvirus with Long-read Single Molecule Real-Time Sequencing

**DOI:** 10.1038/srep43751

**Published:** 2017-03-03

**Authors:** Dóra Tombácz, Zsolt Balázs, Zsolt Csabai, Norbert Moldován, Attila Szűcs, Donald Sharon, Michael Snyder, Zsolt Boldogkői

**Affiliations:** 1 Department of Medical Biology, Faculty of Medicine, University of Szeged, Somogyi B. u. 4., Szeged, H-6720, Hungary; 2 Department of Genetics, School of Medicine, Stanford University, 300 Pasteur Dr., Stanford, CA 94305-5120, USA

## Abstract

Herpesvirus gene expression is co-ordinately regulated and sequentially ordered during productive infection. The viral genes can be classified into three distinct kinetic groups: immediate-early, early, and late classes. In this study, a massively parallel sequencing technique that is based on PacBio Single Molecule Real-time sequencing platform, was used for quantifying the poly(A) fraction of the lytic transcriptome of pseudorabies virus (PRV) throughout a 12-hour interval of productive infection on PK-15 cells. Other approaches, including microarray, real-time RT-PCR and Illumina sequencing are capable of detecting only the aggregate transcriptional activity of particular genomic regions, but not individual herpesvirus transcripts. However, SMRT sequencing allows for a distinction between transcript isoforms, including length- and splice variants, as well as between overlapping polycistronic RNA molecules. The non-amplified Isoform Sequencing (Iso-Seq) method was used to analyse the kinetic properties of the lytic PRV transcripts and to then classify them accordingly. Additionally, the present study demonstrates the general utility of long-read sequencing for the time-course analysis of global gene expression in practically any organism.

The pseudorabies virus (PRV), a neurotropic (alpha-)herpesvirus is an important pathogen of swine and is considered a causative agent of Aujeszky’s disease^1^. PRV is a model organism commonly used to study the molecular pathogenesis of herpesviruses^2^,^3^,^4^, while it is also used for the mapping of neural circuits^5^,^6^,^7^,^8^,^9^ as well as in delivering genetically encoded fluorescence activity markers to the nervous system^10^,^11^.

The lytic cycle of the herpesvirus infection is characterised by the expression of viral genes in an orderly fashion. The PRV genome contains 67 protein-coding genes^12^ belonging to three major temporal groups: the immediate-early (IE), early (E), and late (L) classes^13^,^14^. The immediate early 180 gene (*ie180*), the only IE gene of PRV^15^ [homologous to the ICP4 gene of herpes simplex virus (HSV)^16^], plays an essential role in the transactivation of viral gene expression^17^. The E genes encode proteins necessary for nucleotide metabolism and DNA replication. Other E genes such as the early protein 0 (*ep0*)^18^,^19^ and *ul54* genes^20^ encode transcriptional regulators. The late genes can be subdivided into leaky late (L1 or E/L) and true late (L2 or L) classes, depending upon whether or not DNA replication is required for their expression (such is the case for L2 genes) of these, or not. In addition, the PRV genome also encodes several non-coding transcripts^21^ and transcript isoforms, many of which we identified in our recent study^12^.

The PRV genome is highly compact and contains relatively short intergenic regions. The viral genes are organized into tandem gene clusters encoding polycistronic transcriptional units^22^, which are typical in prokaryotic organisms, but rare in higher-order organisms^23^. However, the polycistronic transcripts of herpesviruses are different from those of prokaryotic transcripts in that the viral genes are expressed in various combinations from the gene cluster and in that the transcripts overlap each other. The typical architecture of an overlapping polycistronic unit can be characterised by a common poly(A) signal (PAS) and varying transcription start sites (TSSs), due to the control by distinct promoters. For example, the following RNA molecules are produced from a tetracistronic unit: 1-2-3-4, 2-3-4, 3-4 and 4, where ‘1’ represents the most upstream gene, while ‘4’ is the most downstream gene. In a recent study, we demonstrated that other gene combinations can also be produced from the polycistronic units, mainly in the form of low-abundance RNA molecules^12^. The downstream genes on a transcriptional unit are thought to be untranslated due to the reason that the eukaryotic RNAs - with some exceptions^24^ - use cap-dependent ribosome-binding sites at their 5′-ends^25^.

The dynamic properties of the herpesvirus transcriptome have been studied using various methods, including microarray^26^,^27^, Illumina sequencing^28^,^29^ and quantitative real-time PCR^30^,^31^ analyses. In this study, PacBio’s Single Molecule, Real-Time (SMRT) sequencing platform was used to investigate the polyadenylated fraction of the PRV transcriptome produced by productive infection of porcine kidney (PK-15) immortalized epithelial cells. The aim of this work was twofold: first, we aimed to characterise and classify the PRV transcripts based on their kinetic properties using a novel method, and second, we strived to demonstrate the utility of long-read sequencing for the quantitative analysis of global transcription, which included the profiling of time-varying gene expression on a genome-wide scale. The most significant advantage of long-read sequencing is its capacity for easy identification of length- and splice transcript isoforms. Additionally, this method is capable of distinguishing the various overlapping mono- and polycistronic RNA molecules. Illumina sequencing and real-time RT-PCR are not efficient in the identification of full-length and complex transcripts, and therefore these techniques are only capable of monitoring the gross activity of genes or of particular genomic regions, but are incapable of conveying which isoforms are expressed at a given time, or whether a certain gene is expressed as a monocistronic transcript or as a part of a polycistronic RNA molecule.

## Results

### Analysis of the time-varying PRV transcriptome using SMRT sequencing data

**Figure 1:**
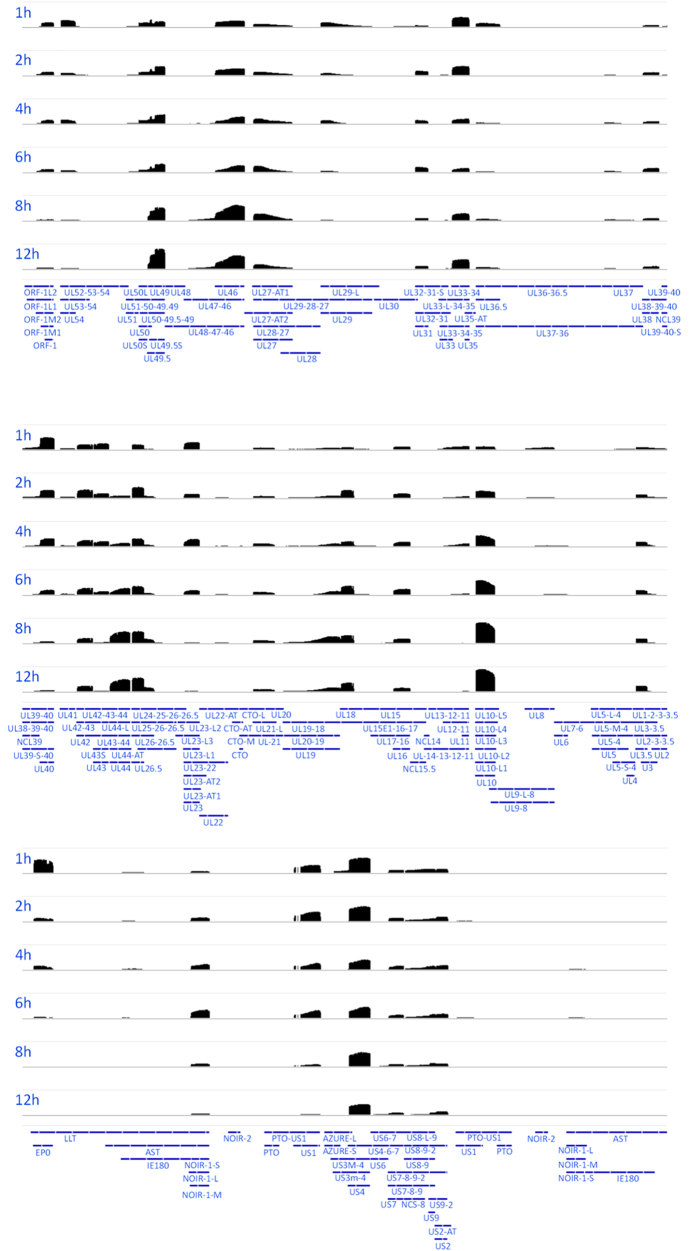
Sashimi plot representation of the transcripts across the PRV genome. The black histograms show normalized coverage values in the samples from 1 h to 12 h p.i., while the transcripts are marked with blue on the genome. The relative coverage of the transcripts change during the replication cycle.

In this study, a PacBio RSII-based polyadenylation sequencing (PA-seq) method was employed to monitor the dynamic profile of the PRV transcriptome on productively infected PK-15 cells. This technique allows a distinction between the mRNAs and the polyadenylated antisense transcripts generated from the same DNA locus. The computational work was carried out by using the SMRT Analysis package. The full-length viral transcripts were quantified by calculation of the reads of inserts (ROIs) obtained from the PRV cDNAs, which were generated by reverse transcription of RNA molecules isolated at various post-infection (p.i.) periods ranging from 1 to 12 hours. Our samples yielded 173,130 ROIs (Supplementary Table 1) from which 57,021 reads aligned to the PRV genome (Fig. 1), while the remainder aligned to the pig genome. The median length of the ROIs was 1244.5 nts. No size-selection was applied in this analysis.

The library preparation and sequencing protocol used in our study [Pacific Biosciences template preparation and sequencing protocol for Very Low Input (10 ng)] favours relatively short (1–2 kb) nucleotide sequences; therefore, the longer and shorter transcripts were underrepresented among the sequencing reads. Although absolute quantification was not possible using our method, we were able to compare the gene expressions by calculating relative expression rates, such as the F_x_ values. Transcripts with average F values smaller than 0.05% were not considered. The polycistronic nature of PRV transcripts presented a further difficulty for the identification of transcripts. The incomplete transcripts produced from a polycistronic unit were assigned to the longer RNA molecules if they contained at least 8 bases from the upstream gene. For explanation, our earlier results^12^ showed a 0 to 4 base pair (bp) length polymorphism at the TSSs of the transcripts, and therefore, we chose the longer 8-bp homology of the 5′-sequences of the ROIs with the putative upstream genes in order to ensure that the given ROI is not only TSS variant, but that it is a different transcript.

### Categorization of transcripts with regard to expression profile kinetics

**Figure 2:**
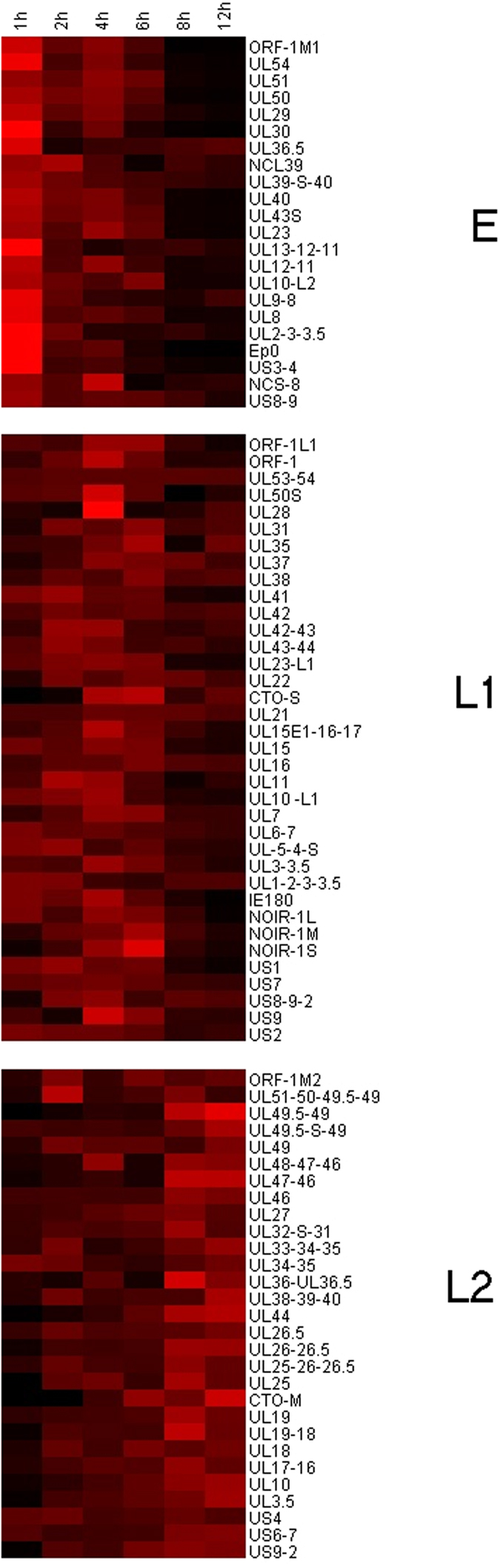
Heat map representation of the transcription kinetics of PRV genes. The F_x_ values of the PRV transcripts show distinct expression profiles in the 3 kinetic clusters. Each row represents the changes of the relative expression of a transcript. Red rectangles indicate high, black rectangles indicate low relative expression values.

**Figure 3:**
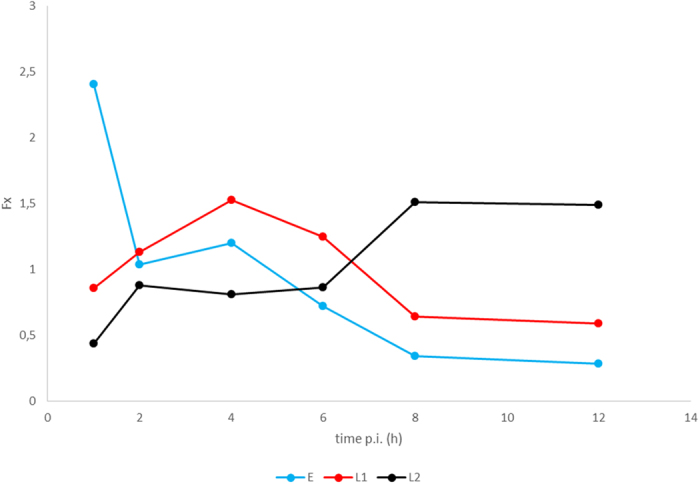
The mean F_x_ values of the different kinetic classes of PRV transcripts. The relative expression rate of early genes is high in the early hours of infection, while those of the late genes are higher in later hours. The L1 transcripts exhibit an intermediate expression profile.

The lytic PRV transcriptome was characterised by the quantification of the polyadenylated RNA molecules produced by the virus during productive infection in cultured cells. The kinetic categorisation of viral transcripts was based on the principle that the E genes are expressed at a high level in the first stage of the viral life cycle, and produce a relatively low amount of gene product later; meanwhile the L2 genes are expressed only at a low level during the first hours of infection, while becoming more abundant later. The L1 genes are characterised by intermediate kinetic profiles. We used k-means clustering of the F_x_ values of the transcripts based on Pearson-correlation to create three clusters (Fig. 2). Figure 3 shows that the overall E, L1 and L2 gene expressions significantly differ from one another. E genes exhibit high relative expression prior to the onset of DNA replication, which by 12 h p. i. then declines considerably. The L2 genes exhibit inverse kinetics compared to those of E genes. The L1 genes behave differently than the E genes at the beginning of infection, while later the expression curve of these became similar. To the contrary, L1 genes display expression dynamics similar to those of L2 genes during the first hours of infection, which becomes different during the second half of infection. The ratio of E gene products in comparison to their maximal values was higher than in the L genes during the first 4 h of infection. Independently of the kinetic classes, the amounts of gene products increase between 6 to 8 h p. i.; apparently due in part to the multiplication of the viral DNA molecules. The amounts of E gene products typically decrease around 8 and 12 h, which was contrary to the increasing quantity of L2 transcripts within this time period.

**Figure 4:**
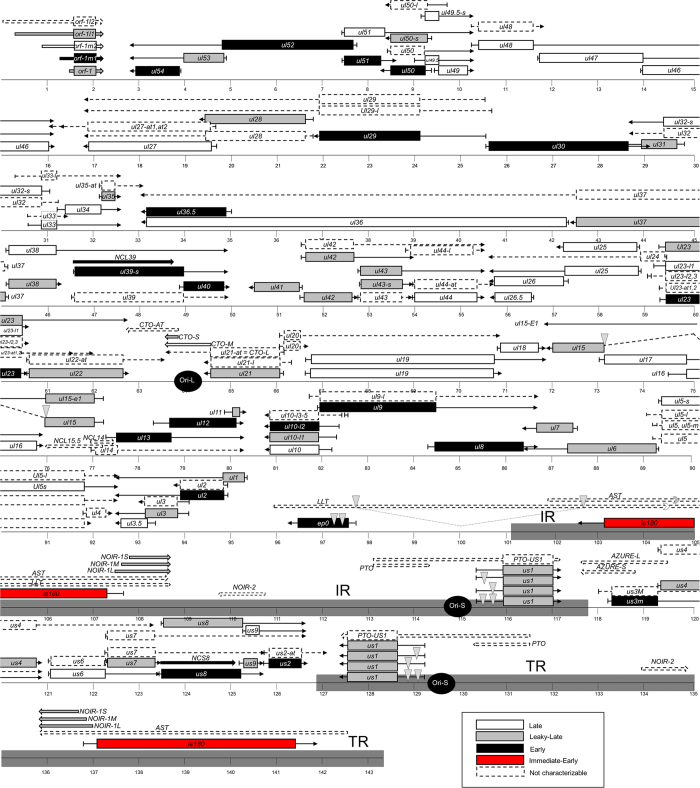
Kinetic map of the PRV transcriptome. Transcripts in white belong in the late, grey belong in the leaky-late, black in the early, and red in the immediate-early classes. Transcripts illustrated with dashed boxes and lines represent relatively low read counts. Boxes represent the first ORF on an RNA molecule, while arrows without boxes are non-coding sequences. Introns are marked by grey triangles.

We also characterised the novel transcripts reported in our recent publications^12^,^32^. Supplementary Figure 1 shows the A6 values of these transcripts at different p.i. periods. In our kinetic characterisation, we classified UL36.5 and ORF-1M1 as early, while ORF-1L1, ORF-1, CTO-S and all isoforms of NOIR-1 were found to show leaky-late kinetics, and CTO-M and ORF-1M2 were clustered into the true late group. It is worth noting that the beginning of expression of the CTO-M transcript that overlaps the OriL coincides with the onset of viral DNA replication. This finding adds further support to the hypothesis that the OriS-RNA in HSV^33^ and the CTO transcripts^34^ may have important roles in the regulation of DNA-replication. The recently identified UL36.5 is a truncated version of the UL36 transcript. The longer UL36 transcript has late kinetics, while the shorter UL36.5 was found to exhibit early transcription kinetics. UL36 is a tegument protein of the PRV, having a role in the viral egress^35^. To date, no protein that is translated from the UL36.5 transcript has been described. Sixty amino acids at the C terminus of the (p)UL36 have been shown to be highly conserved among α-herpesviruses and considered essential to the PRV^36^, however the majority of the protein coded by the UL36.5 region has been found to be non-essential in the mutational analyses^37^. Of the 147 described transcript isoforms of the PRV, 87 isoforms had produced ROIs sufficient for clustering. Figure 4 shows a map depicting the location of the genes coding for the characterised transcripts on the genome, along with their kinetic classes.

### DNA replication exerts a global repression on transcription

**Figure 5:**
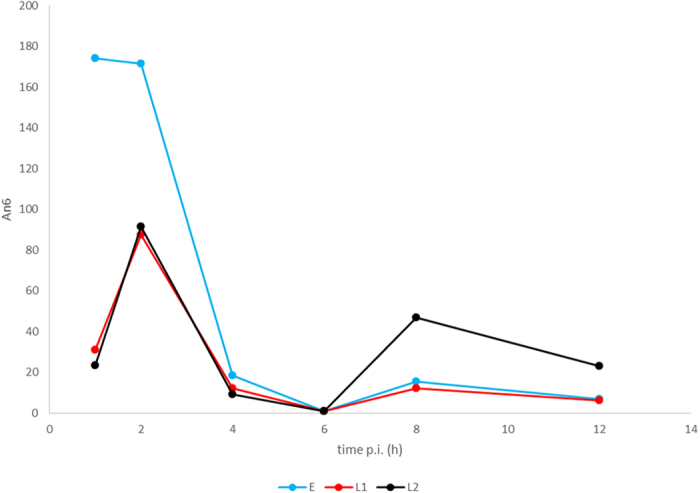
Transcription rate per genome drops substantially at 4 h p.i. The graph shows the mean An6 values of the different kinetic groups. When the viral replication starts, the expression of transcripts in all three kinetic classes drops and only true late transcripts retain a relatively high expression rate from individual DNA molecules.

While global gene expression evidently rose steeply from 6 h p.i., the number of RNAs normalised to the relative copy number of viral DNA had substantially decreased from 4 h p.i., which is the onset of the replication of PRV genome (Fig. 5, Supplementary Table 2). The data show that even the expression of L2 genes is significantly reduced from a single DNA molecule. There is an approximately 100-fold difference at 6 hour post infection. These data confirm our earlier results that were obtained by RT^2^-PCR analysis^38^. Apparently, this phenomenon is related to the DNA replication. Whether the cause for the drastic fall in gene expression rate is due to the interference between the transcription and replication machineries or due to something completely different is open to speculation.

### Comparison of the Pac-Bio and RT-qPCR-based classifications

In this study we re-evaluated our earlier RT-qPCR data^30^ using the same method (k-means clustering of the F_x_ values) that we used to analyse the PacBio data (Supplementary Figure 2). For comparison of our results in the two experiments we considered only the genes with transcript isoforms belonging to only one kinetic class, as with the PacBio data. As Table 1 shows the two methods produced similar results: less than one third of the genes were classified differently. Most of these cases were L1–L2 changes of kinetic classes, for which the major reason was that the qPCR methods produced a higher L1/L2 ratio than those of the long-read sequencing platform. The discrepancies in the results may also be explained by the fact that the RT^2^-PCR approach characterises the gross transcriptional activity of genetic loci and thus could not make a distinction between particular transcript isoforms and mono- and polycistronic expression of genes. This distinction is also important because downstream genes in polycistronic transcription units are likely to be untranslated. A further difference in the two approaches is that during the qPCR analysis a low (MOI = 0.1) titre of viruses was applied, while in this study we used a high (MOI = 10) dose of viruses for the infection. We had shown in an earlier study that there are differences in the transcription dynamics of many genes between low- and high-titre infections^31^. Our aim with this comparison was to demonstrate that despite the different conditions used in the two experiments, the results obtained are indeed similar.

### Correlation of gene expressions with the transcription of the IE180 transactivator

**Figure 6:**
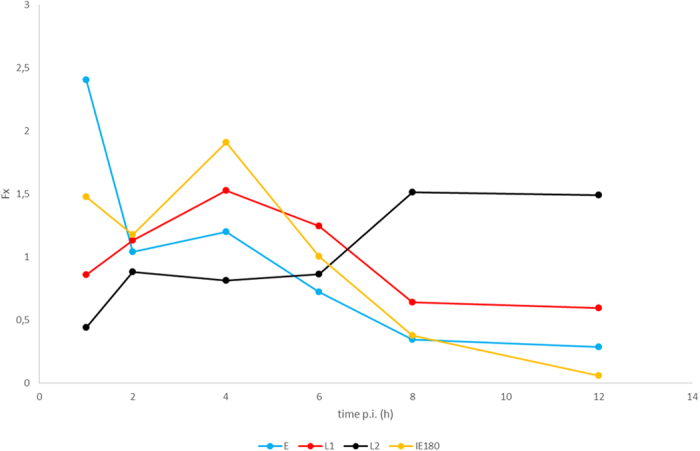
The F_x_ values of the IE180 transcript compared to other kinetic classes. The expression profile of IE180 transcript resembles to those of L1 genes.

The IE transcripts of herpesviruses are expressed shortly after infection^39^. The only immediate-early gene of PRV is the *ie180*, the protein product of which is a major coordinator of gene expression^40^. Being an IE gene the relative expression of this gene is expected to peak during the first hour of infection. Since our first sample was isolated 1 h after infection, we were not able to characterise a distinct IE class of transcripts. However, previously we were able to show that the expression of the *ie180* gene is independent of *de novo* protein synthesis^30^, which is a characteristic of immediate-early herpes virus genes. High positive correlation was detected between the levels of IE180 transcripts and the levels of both the E (0.72) and L1 (0.83) gene products, and an inverse correlation between the transcripts from the transactivator and the L2 genes (−0.86) within the 1 to 12 h infection period (Fig. 6). The differential correlation of IE180 protein with the different kinetic classes of PRV genes could possibly be explained by the finding that the ICP4 transactivator of HSV, homologous to the IE180 protein, can either increase or decrease the rate of transcription mediated by the host RNA polymerase, depending on the target promoter^16^, and that the study of temperature-sensitive ICP4 mutants revealed a differential effect of this transcription factor on the early and late gene expression^41^ (these mutants express significant levels of early polypeptides, but not late gene products). Theoretically, there are three possible effects that can explain the observed phenomenon. In the first scenario, the IE180 protein directly determines the levels of gene products by exerting a stimulatory effect on the E genes and a repressive effect on the L2 genes. Similarly to ICP4^42^, the IE180 protein also exerts a self-inhibitory effect^43^ at the late stage of infection, which might explain the high level of L2 genes that are present at the late period of infection. Alternatively, IE180 can only facilitate the switch between E and L genes, as assumed in the case of the ICP4^41^ and the inverse expression profile between the IE180 and the L2 genes is a mere coincidence in this scenario. A third possibility might be a reverse causation between the IE180 and the L2 proteins as certain L proteins are assumed to down-regulate the transactivator in HSV^41^.

### Transcriptional kinetics of cistron variants

The polycistronic transcription units can be controlled by multiple promoters. Here we analysed the transcripts variants (Supplementary Figures 3–7) that contained varying numbers of genes and share a common PAS (5′-cistron variants). As a result, we established that 8 pairs of the 5′-cistron variants showed similar expression kinetics (Pearson correlation coefficient > 0.5), and that 9 of them displayed inverted kinetics (Pearson correlation coefficient <−0.5), while an additional 14 pairs were expressed differentially (Pearson correlation coefficient ≤0.5, but ≥−0.5). A promoter can control the transcription of multiple 3′-cistron variants, including those containing one or more genes. Using long-read sequencing, we were able to characterise each of these types of variants, which had not been possible using RT-PCR or short-read sequencing. We found that 4 pairs of the 3′-cistron variants that share common promoters exhibited similar expression kinetics, while another set of 4 pairs produced differential kinetics.

### Transcriptional kinetics of UTR isoforms

We also analysed those 5′-UTR variants that are controlled by distinct promoters, but contain the same number of genes, and 3′-UTR isoforms with the same genes, but with distinct PASs. We obtained results indicating that the 5′-UTR isoforms displayed similar expression kinetics in 10 gene pairs, while these were expressed with differential kinetics in three genes. A common feature of the exceptions, the *orf-1* and *ul10* genes, is that they have multiple TSSs (5 or 6, respectively; Supplementary Figures 8–11), while *ul36* produces a different polypeptide from the *ul36.5* gene. We further established that both of the 3′-UTR isoforms which share the same promoters displayed similar expression kinetics.

### Transcriptional kinetics of splice isoforms

We examined whether the proportions of the different splice variants of UL15, EP0 and US1 transcripts change during the time course of infection (Table 2). We were able to establish that the ratio of the non-spliced UL15 transcript (UL15E1-16-17) did not change compared to the spliced isoform throughout the duration of infection.

The ratio of the isoform-1 (intron length: 138 nts) to non-spliced variant of EP0 transcript was approximately 25% at the first 2 hours of infection. This percentage dropped to 10.8% by 4 h, but increased to 46.8% by 6 h p.i. Isoform-2 (intron length: 90 nts) was expressed to a low degree throughout the entire replication cycle. We obtained a few ROIs from the lowest-abundance EP0 transcripts from later infection periods, and therefore we were not able to establish any change in its ratio in comparison to the more abundant ones.

The *us1* gene produces four splice variants, as follows: US1–1 (intron length: 120 nts), US1–2 (intron length: 156 nts), US1–3 (both introns are spliced out), and a non-spliced transcript. The double-spliced variant was transcribed in the largest proportion by far (~99%). There were no substantial changes in the ratio of splice isoforms throughout the examination period. The non-spliced and partially spliced variants were expressed in a very low amount; therefore, these may represent incompletely processed RNA molecules. This may indicate that only the double-spliced isoform may have a function.

The biological function of differential expression of the splice isoforms has not been established in this study.

## Discussion

The analysis of temporally changing transcription profiles is valuable for understanding the regulation of gene expression on a genome-wide scale. In comparison to cellular organisms, viruses only have a small number of genes and therefore, can be ideal models for both the comprehensive analysis of the dynamic transcriptome, as well as in testing the utility of long-read sequencing for this purpose.

We have characterised the dynamic transcriptome of the PRV using a long-read sequencing method. Comparing our current results to our RT-qPCR classifications, we were able to obtain results that differed only slightly, however with long-read sequencing we were able to differentiate between many transcript isoforms that had been indistinguishable by RT-qPCR. In this study, we utilised oligodT-based reverse transcription for the non-amplified Iso-Seq method, which allows for us to determine whether a transcript is produced from the plus or minus DNA strand. In a recent study^12^; we were able to demonstrate that in practice, both DNA strands are actively transcribed across the entire viral genome. Most of the antisense RNA sequences are assumed to be produced from the promoters of adjacent or distal convergently-oriented genes. Since these complex transcripts, which contain both sense and antisense sequences are very long, in most cases we were not able to identify their TSSs (with the exception of, for example, the UL50-49.5-49 transcript: see Fig. 4). We were however able to detect antisense and non-coding polyadenylated RNAs, which are controlled by their own promoters, such as the various CTO, NOIR, AZURE, and PTO transcripts. Nevertheless, some of these novel RNA molecules occur either in very low copy numbers, or are either too short or too long and therefore we were not able to classify all of these even using PacBio sequencing.

Our results have revealed that even though the throughput of long-read RNA sequencing techniques available today is significantly lower than that of the techniques based on short-read sequencing; it can be efficiently used to quantify the total transcripts of an organism, including a dynamic transcriptome. Long-read RNA-sequencing is considered to be a method superior to RT^2^-PCR, microarray studies, and short-read RNA-sequencing in that it is able to distinguish between transcript isoforms and overlapping transcripts.

The similarity of gene expressions of 3′-UTR isoforms and 3′-cistron variants comes as no surprise, as these are controlled by the same promoters. We also observed similar transcription patterns in 5 out of 7 5′-UTR isoforms, and in 12 out of 21 5′-cistron variants, all of which suggest that these genes are driven by promoters that provide similar kinetic properties. Nevertheless, a higher resolution of infection cycle may reveal differences in the expression of these genes. We found that 5 out of 9 3′-cistron variants exhibited different transcription kinetics. The potential reason for this phenomenon may be that the transcriptional machinery could somehow change throughout the progression of the viral life cycle, and therefore could exert distinct effects on the same promoter thereby producing different kinetics for the cistron variants.

Due to the relatively low throughput of long-read sequencing, the low-coverage transcripts do not produce statistically reliable results, and therefore they were not included in the kinetic analysis. DNA replication leads to an increase in the copy number of the viral DNA, which may create difficulties in the comparison of gene expression at different time periods of infection. Furthermore, we observed a global suppression on gene expression in individual DNA molecules following the onset of replication; this in turn makes the analysis of the dynamic transcriptome more difficult.

Our PA-Seq method may underestimate the amount of long and short transcripts due to the preference for sequences within the range of 1 to 2 kbps during library preparation and sequencing. Furthermore, the long polycistronic transcripts are underestimated due to the inefficiency of reverse transcription over long distances from the poly(A) tail and the disintegration of long RNA molecules due to the RNase activity of the virion host shut-off protein of PRV^44^. In order to eliminate the problems outlined above, we used dimensionless relative values for the analysis of the transcription kinetics.

Similarly to our RT^2^-PCR results^30^ in this study, we were able to establish that all promoters in the PRV genome are active at all times but that they differ in the frequency with which the transcription is initiated.

In conclusion, in this study we demonstrated the utility of long-read sequencing for the investigation of the dynamic transcriptome of a herpesvirus. We have established that this technique can also be applied in the study of processes exhibiting a definite, well-controlled time-course of transcription, such as during viral replication, embryogenesis, tissue regeneration, etc. Furthermore, we succeeded in monitoring the individual dynamics of transcripts isoforms, intron variants and overlapping RNAs, which are especially important in herpesviruses producing complex transcriptome profiles throughout the productive infection. Additionally, we have characterised the kinetic properties of several novel PRV transcripts.

## Materials and Methods

### Cells and virus

Monolayer cultures of porcine kidney (PK-15) cells were used for the propagation of the wild-type pseudorabies virus (PRV, strain Ka). PK-15 cells were cultivated in Dulbecco’s modified Eagle medium supplemented with 5% foetal bovine serum (Invitrogen) with 80 *μ*g gentamycin/ml at 37 °C in the presence of 5% CO_2_. The virus stock was prepared as follows: rapidly-growing semi-confluent PK-15 cells were infected at a multiplicity of infection (MOI) of 10 plaque-forming unit (pfu)/cell followed by incubation until a complete cytopathic effect could be observed. The infected cells were then frozen, thawed three times, and centrifuged at 10,000 g for 15 minutes. The titre of the virus stock was determined in the same cell line. For the PacBio analysis, cells were infected with a high MOI (10 pfu/cell), while for the RT^2^-PCR analysis we used low-titre (MOI = 0.1) infection. In both cases infected cells were incubated for 1 h at 37 °C, followed by removal of the virus suspension and washing the cells with phosphate-buffered saline. After the addition of new medium to the cells, they were incubated for 1, 2, 4, 6, 8, or 12 h.

### Pacific Biosciences RS II sequencing

Total RNA was isolated from virus-infected epithelial cells using the NucleoSpin^®^ RNA kit (Macherey-Nagel). Polyadenylated RNAs were purified from 40 μg total RNA using the Oligotex mRNA Mini Kit (Qiagen), according to the manufacturer’s recommendations for the Oligotex mRNA Spin-Column Protocol. The isolated PolyA(+) fractions were quantified with the Qubit RNA HS Assay Kit (Life Technologies), and then were converted to cDNAs using 0.2 μg RNA, Anchored Oligo(dT) 20 primers (Life Technologies) and the SuperScript Double-Stranded cDNA Synthesis Kit (Life Technologies; the included SuperScipt II enzyme was changed to SuperScript III). The obtained double-stranded cDNAs were quantified with the Qubit HS dsDNA Assay Kit (Life Technologies). SMRTbell templates were generated from cDNAs (80 ng each) with the PacBio DNA Template Prep Kit 1.0 following the Pacific Biosciences template preparation and sequencing protocol for Very Low (10 ng) Input 2 kb libraries with carrier DNA (pBR322, Thermo Scientific). SMRT bell libraries were bound to DNA polymerases with the DNA/polymerase binding kit P4 and v2 primers. The obtained DNA polymerase/template complexes were bound to MagBeads using the Pacific Biosciences MagBead Binding Kit. The DNA-sequencing was carried out on the Pacific Biosciences RSII platform with C3 sequencing reagents. The movie length was 180 min long. The quality of the libraries was analysed using the Agilent 2100 Bioanalyzer.

### Reverse-transcription real-time PCR

RT-qPCR was performed as described in our previous publication^30^. Briefly, total RNA was isolated from the cells at various times after viral infection (1 h, 2 h, 4 h, 6 h, 8 h) using the Macherey-Nagel’s NucleoSpin^®^ RNA kit. The samples were treated with TURBO DNA-free™ Kit (Life Technologies) to remove residual genomic DNA. The RNA quantity was measured by Qubit 2.0 with Qubit^®^ RNA BR Assay Kit. The RNA was reverse-transcribed into cDNA with SuperScript III (Invitrogen) using 70 ng total RNA and strand-specific primers. Potential DNA contamination was excluded using no-RT controls. Quantitative Real-Time-PCR experiments were carried out with a Rotor-Gene 6000 cycler (Corbett Life Science) in 20 μl final volume using Absolute QPCR SYBR Green Mix (Thermo Scientific) containing 7 μl of cDNA solution (diluted 10-fold), 1.5 μl of forward and 1.5 μl of reverse primers (10 μM each). The running conditions were 15 min at 95 °C, followed by 30 cycles of 94 °C for 25 sec (denaturation), 60 °C for 25 s (annealing), and 72 °C for 6 s (extension). Amplification specificity was confirmed by a melting-curve analysis following the PCR.

### Analysis of the long-read sequencing data

Read processing was carried out with SMRT Analysis v2.3.0. Reads were aligned to both the swine genome (*Sscrofa10.2*) and the PRV genome (KJ717942.1)^45^ using GMAP^46^ with the following parameters: gmap —npaths 0 —format samse -H 30 -K 3000 -L 3000 —min-intron length 40. The mapped reads were assigned to transcript isoforms if the read coordinates did not exceed the 5′ or 3′ ends of the isoforms by more than 7 nts. The direction of the reads was determined based on the polyA-sequence. If the read could be matched to more than one isoform, then it was assigned to the shortest one. We normalised the read count to the mitochondrial reads (A values) and also to the relative copy number of PRV DNA (A_n_ values). The frequency (F value) of each transcript isoform was calculated by dividing the read count of the transcript by the total viral read count in the sample. Relative expression values were calculated by normalising the F values of each transcript to the mean F values of the given transcript (F_x_ values).

### Analysis of the RT-qPCR data

R values of a gene at given time points were calculated by the E ^ (−Ct) method using the mean E ^ (−Ct) value of all measured viral genes in a biological sample as reference. The R value of a gene divided by the mean R values of that gene at different time points, results in the same relative expression values as described earlier (F_x_ values).

F, Fx, A, An, A6 and An6 values were calculated according to the following equations:


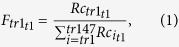



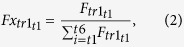



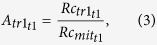



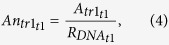



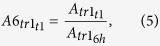



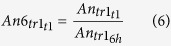


where R_c_ is the number of reads assigned to a transcript (tr), t is a given time post infection Rc_mit_ is the number of the mitochondrial transcripts. R_DNA_ is the relative amount of DNA, measured.

### Kinetic classification

Transcript isoforms with an average F value higher than 0.05% were considered for kinetic classification. Isoforms were clustered into 3 groups according to their relative expression values by k-means clustering with Euclidean distance matrix, using the Gene Cluster 3.0 program^47^. The group of transcripts with high relative expression values in the first hours following infection was considered the early (E) cluster, the transcripts with high relative expression values in the later stages of infection were characterised as late (L2), and transcripts outside these clusters were labelled leaky-late (L1).

### Normalization of PRV transcripts with mitochondrial ROIs and DNA copy number

To calculate the A values (and the derived A_n_ values) of the transcripts, we normalized the PRV reads to mitochondrial read counts, aligning to the *Sus scrofa* 10.2 MT chromosome sequence. The degradation of RNAs during alpha herpesvirus infection^48^ poses a severe problem for quantitative analysis of the dynamic transcriptome, because of the difficulties in the selection of reliable reference RNA molecule(s). We use the 28 S rRNA molecule in our real-time RT-PCT studies, but it is not applicable for PA-seq analysis. A study has shown that mtDNA in HSV-1-infected Vero cells decrease due the UL12.5 gene of HSV-1^49^. mtRNAs were chosen as references because the PRV does not express a UL12.5 gene product.

The degradation of RNAs during alpha herpesvirus infection^48^ poses a severe problem for quantitative analysis of the dynamic transcriptome, because of the selection of the difficulties of the selection of reliable reference RNA molecule(s). We use the 28 S rRNA molecule in our real-time RT-PCT studies, but it is not applicable for PA-seq analysis.

### Data availability

Reads mapped to the swine genome (*Sscrofa10.2*) and the PRV genome (KJ717942.1) are available at the European Nucleotide Archive (http://www.ebi.ac.uk/ena) under the accession numbers ERR1725020, ERR1725021, ERR1725022, ERR1725023, ERR1725024, and ERR1725025

## Additional Information

**How to cite this article**: Tombácz, D. *et al*. Characterization of the Dynamic Transcriptome of a Herpesvirus with Long-read Single Molecule Real-Time Sequencing. *Sci. Rep.* **7**, 43751; doi: 10.1038/srep43751 (2017).

**Publisher's note:** Springer Nature remains neutral with regard to jurisdictional claims in published maps and institutional affiliations.

## Supplementary Material

Supplementary Information

## Figures and Tables

**Table 1:** Comparison of the RT-qPCR and the long-read sequencing data.

	RT-qPCR	Long-read sequencing
ul53	L1	L1
ul54	E	E
ul49.5	L2	L2
ul48	L1	L2
ul47	L1	L2
ul46	L2	L2
ul29	E	E
ul28	L1	L1
ul27	L2	L2
ul30	E	E
ul32	L1	L2
ul31	L1	L1
ul33	L1	L2
ul34	L1	L2
ul35	L1	L1
ul37	L1	L1
ul39	L1	E
ul40	E	E
ul41	L1	L1
ul42	L1	L1
ul44	L2	L2
ul25	L2	L2
ul26	L2	L2
ul21	L1	L1
ul22	L1	L1
ul19	L1	L2
ul18	L2	L2
ul15	L1	L1
ul17	L2	L2
ul16	L1	L1
ul13	E	E
ul12	L1	E
ul11	E	L1
ul9	E	E
ul8	E	E
ul6	L1	L1
ul7	L1	L1
ul1	L1	L1
ul2	E	E
ul3	E	L1
ul3.5	L2	L2
us3	E	E
us4	L1	L2
us6	L1	L2
us7	L1	L1
us2	L1	L1
us1	E	L1
ep0	E	E

Transcripts were clustered according to their F_x_ values from the RT-qPCR and long-read sequencing data. Genes are only listed if all their transcript isoforms were clustered into the same kinetic class.

**Table 2:** Splice variants.

(a)	UL15E1-16-17	UL15		
1 h	5 (19.23%)	21 (80.77%)		
2 h	13 (30.95%)	29 (69.05%)		
4 h	5 (38.46%)	8 (61.54%)		
6 h	16 (30.19%)	37 (69.81%)		
8 h	6 (42.86%)	8 (57.14%)		
12 h	5 (33.33%)	10 (66.67%)		
(**b**)	**EP0-1**	**EP0-2**	**EP0-in**	
1 h	129 (24.48%)	18 (3.42%)	380 (72.11%)	
2 h	53 (23.45%)	3 (1.33%)	170 (75.22%)	
4 h	4 (10.81%)	0 (0.00%)	33 (89.19%)	
6 h	29 (46.77%)	1 (1.61%)	32 (51.61%)	
8 h	0 (0.00%)	0 (0.00%)	6 (100.00%)	
12 h	2 (33.33%)	0 (0.00%)	4 (66.67%)	
(**c**)	**US1-both**	**US1-1**	**US1-2**	**US1-in**
1 h	246 (98.01%)	1 (0.40%)	1 (0.40%)	3 (1.20%)
2 h	234 (92.86%)	7 (2.78%)	2 (0.79%)	9 (3.57%)
4 h	31 (100.00%)	0 (0.00%)	0 (0.00%)	0 (0.00%)
6 h	189 (97.93%)	0 (0.00%)	2 (1.04%)	2 (1.04%)
8 h	53 (100.00%)	0 (0.00%)	0 (0.00%)	0 (0.00%)
12 h	37 (97.37%)	1 (2.63%)	0 (0.00%)	0 (0.00%)

The table shows the number of the reads for each splice variant and their percentage in parentheses at different p.i. times. (a), (b), and (c) show the changes in the amount of the splice variants of the UL15, EP0 and US1 transcripts, respectively.
