# Supplementary material for: Characterization of the Dynamic Transcriptome of a Herpesvirus with Long-read Single Molecule Real-Time Sequencing
Source: Sci Rep. 2017 Mar 3;7:43751. doi: 10.1038/srep43751 (PMC5335617; doi:10.1038/srep43751)
Supplement: Supplementary Information [file srep43751-s1.docx]

**Characterization of the Dynamic Transcriptome of a Herpesvirus with Long-read Single Molecule Real-Time Sequencing**

Dóra Tombácz^1†^, Zsolt Balázs^1†^, Zsolt Csabai^1^, Norbert Moldován^1^, Attila Szűcs^1^, Donald Sharon^2^, Michael Snyder^2^, Zsolt Boldogkői^1*^

*Addresses*:

^1^Department of Medical Biology, Faculty of Medicine, University of Szeged, Somogyi B. u. 4., Szeged, H-6720, Hungary

^2^Department of Genetics, School of Medicine, Stanford University, 300 Pasteur Dr., Stanford, CA 94305-5120, USA

^*^Corresponding author

E-mails:

[tombacz.dora@med.u-szeged.hu](mailto:tombacz.dora@med.u-szeged.hu)

balazs.zsolt@med.u-szeged.hu

[csabai.zsolt@med.u-szeged.hu](mailto:csabai.zsolt@med.u-szeged.hu)

[moldovan.norbert@med.u-szeged.hu](mailto:moldovan.norbert@med.u-szeged.hu)

szucs.attila.1@med.u-szeged.hu

[dsharon@stanford.edu](mailto:dsharon@stanford.edu)

[mpsnyder@stanford.edu](mailto:mpsnyder@stanford.edu)

[boldogkoi.zsolt@med.u-szeged.hu](mailto:boldogkoi.zsolt@med.u-szeged.hu)

^†^these two authors contributed equally to this work

^*^Corresponding author

**Supplementary Figures and Tables**

|  | **Swine** | **PRV** | **Unmapped** | **Total** |
| --- | --- | --- | --- | --- |
| **1h p.i.** | 57566 | 7466 | 2672 | 67704 |
| **2h p.i.** | 25130 | 13068 | 1100 | 39298 |
| **4h p.i** | 2755 | 2392 | 158 | 5305 |
| **6h p.i.** | 20000 | 12295 | 1044 | 33339 |
| **8h p.i.** | 1888 | 8134 | 125 | 10147 |
| **12h p.i.** | 3472 | 13666 | 199 | 17337 |
| **Total** | 110811 | 57021 | 5298 | 173130 |

**Supplementary Table 1. The numbers of reads of inserts (ROIs) aligning to the swine and the PRV genomes.**

| **A_n_** | **2h/4h** | **2h/6h** | **2h/8h** | **2h/12h** |
| --- | --- | --- | --- | --- |
| **E** | 8.32 | 105.83 | 15.42 | 38.80 |
| **L1** | 10.81 | 76.05 | 6.86 | 15.01 |
| **L2** | 11.94 | 76.19 | 2.50 | 4.59 |

**Supplementary Table 2. The proportion of the amount of average PRV transcripts produced from a single DNA molecule at various times of infection compared to the 2h p.i. values.** This Table shows that the transcriptional activity from a single viral genome is significantly reduced in every kinetic class of genes following the onset of DNA replication


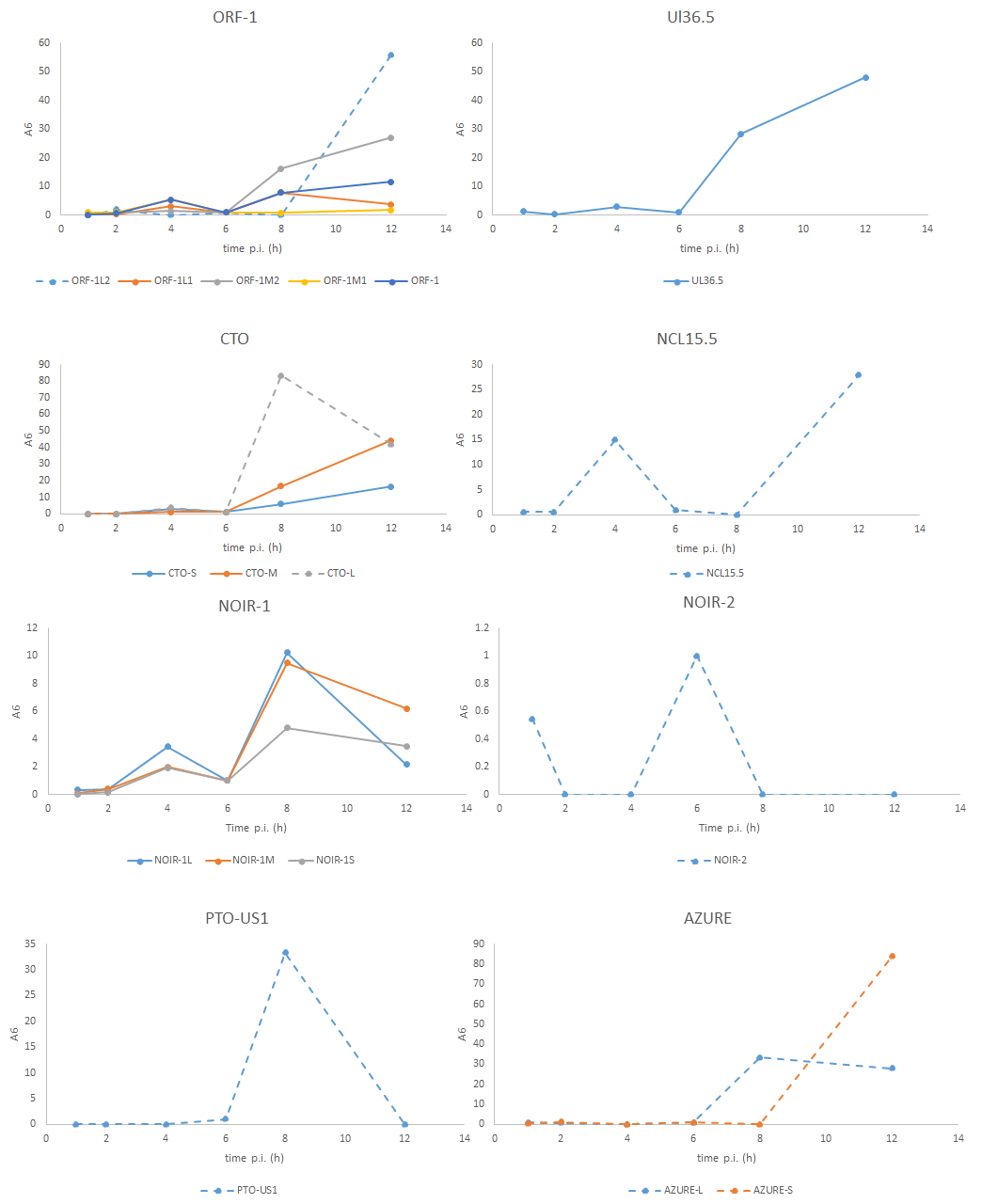


**Supplementary Figure 1. The expression profiles of novel transcripts and transcript isoforms based on the A6 values**. Transcripts with an average F value lower than 0.05% are represented by dashed lines.


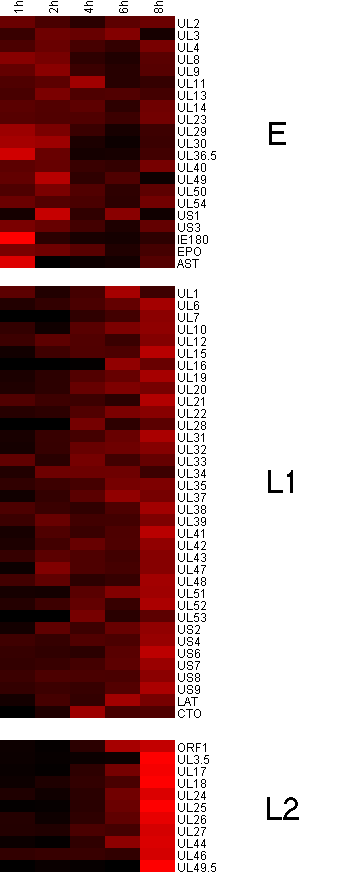


**Supplementary Figure 2 Heat map representation of the transcriptional kinetics measured by RT-qPCR.** The PRV transcripts were clustered according to their F_x_ values by k-means clustering, using Euclidean distance similarity metrics. Red rectangles indicate high, black rectangles indicate low relative expression values.


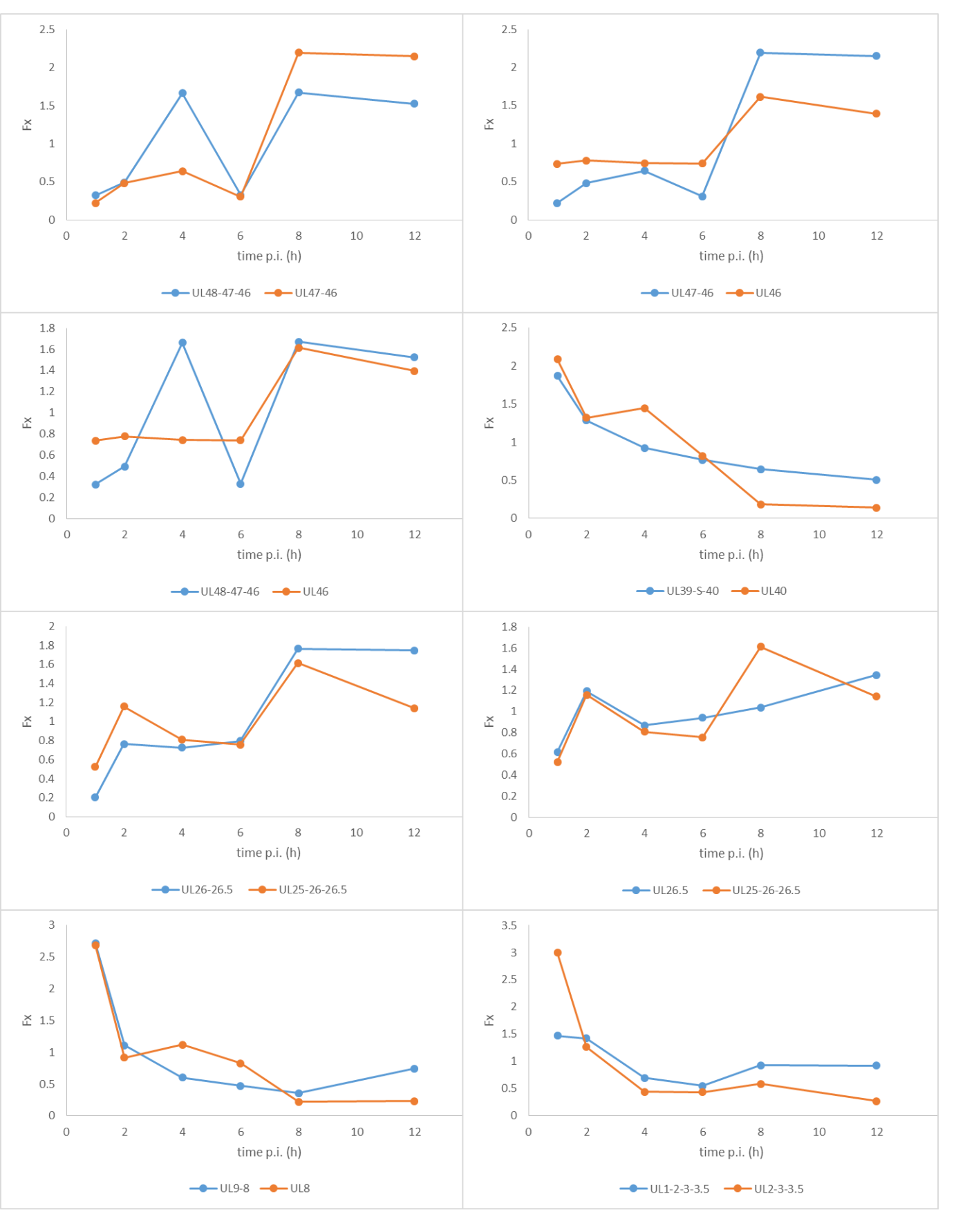


**Supplementary Figure 3. 5’ cistron variant pairs that showed similar expression kinetics (Pearson correlation coefficient >0.5).** The 5’ cistron variants have a common polyA-site, but contain a different number of cistrons.


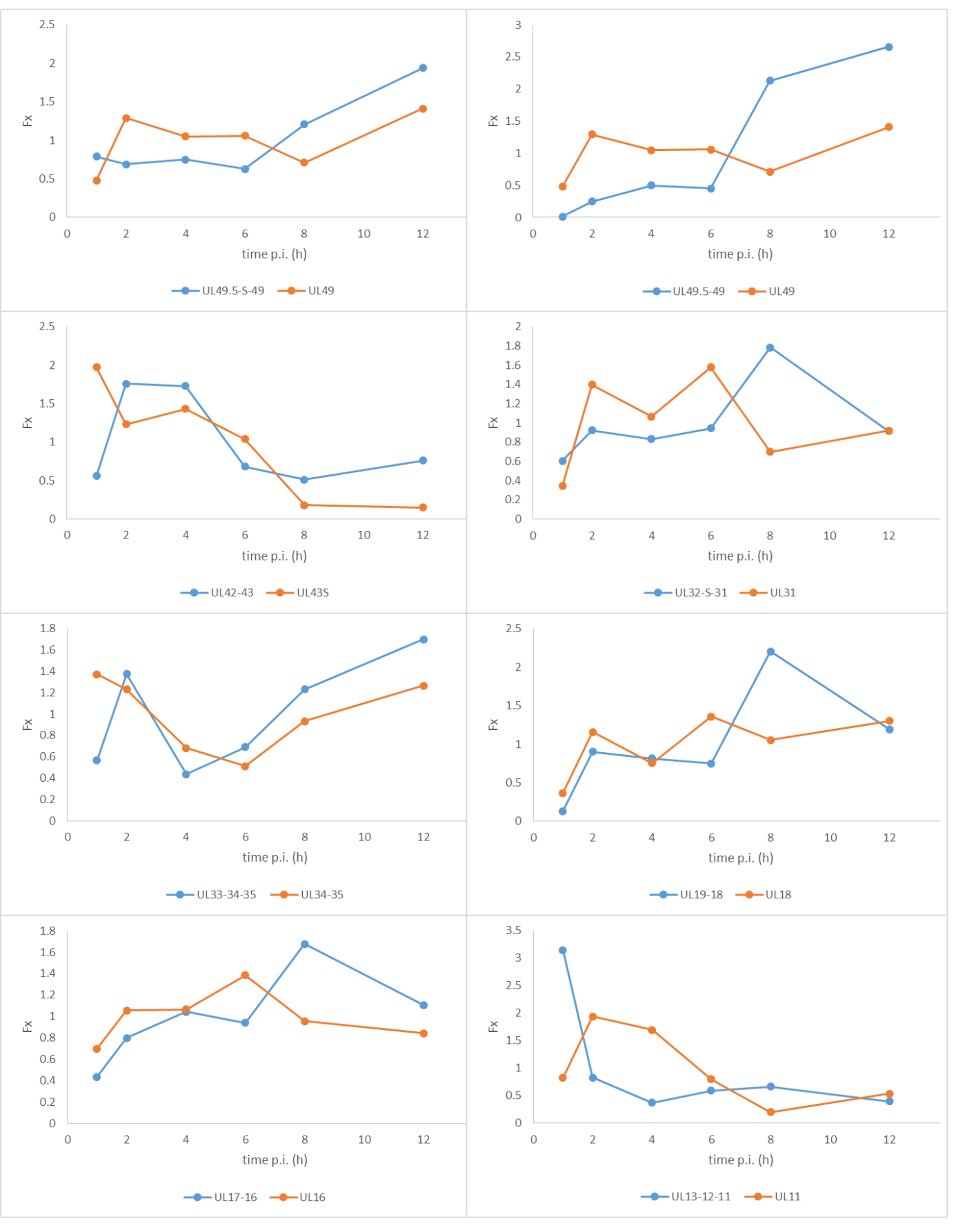


**
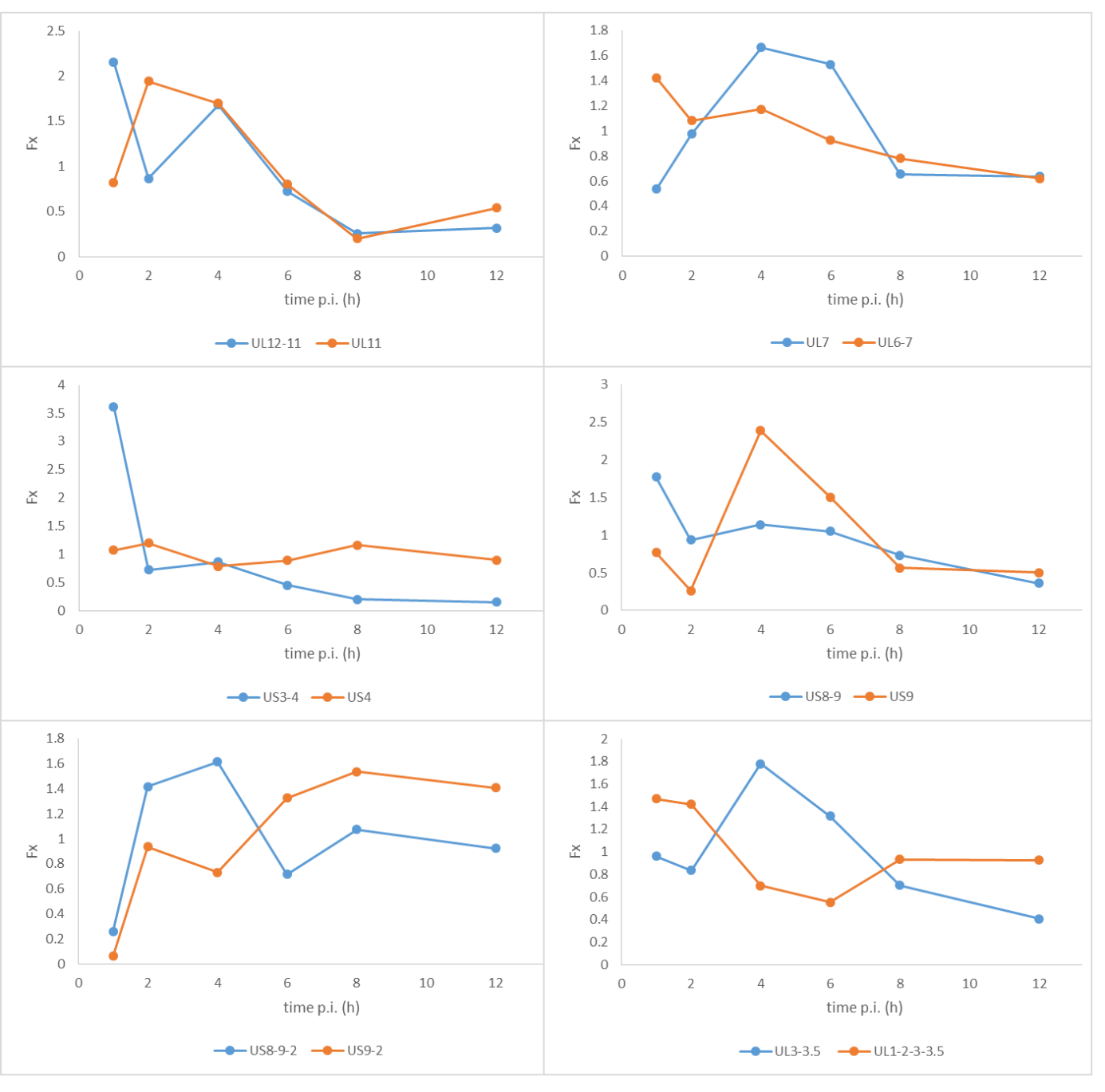
**

**Supplementary Figure 4. 5’ cistron variant pairs showing different expression kinetics (Pearson correlation coefficient ≤0.5, but ≥-0.5).** The 5’ cistron variants have a common polyA-site, but contain a different number of cistrons.


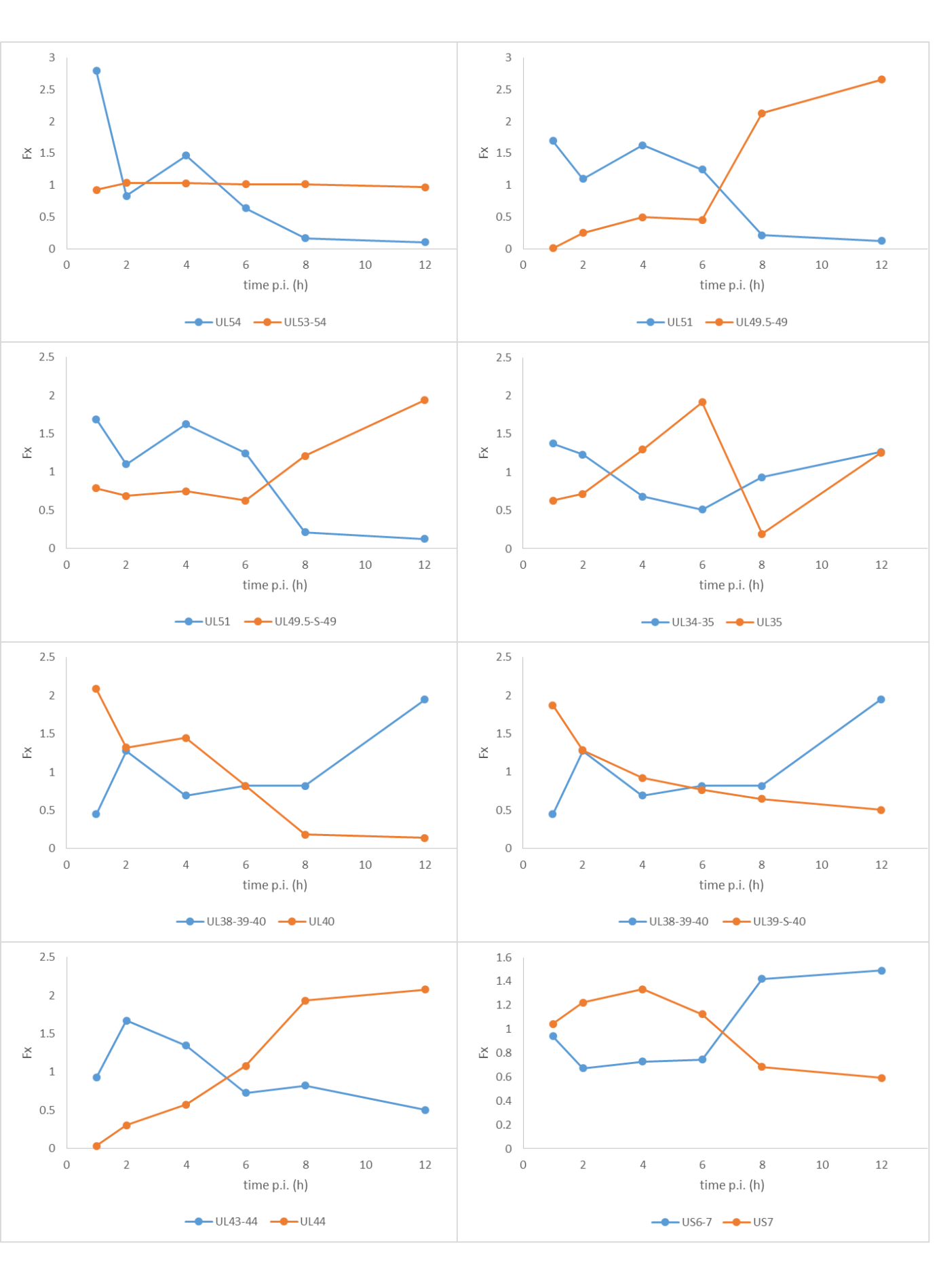


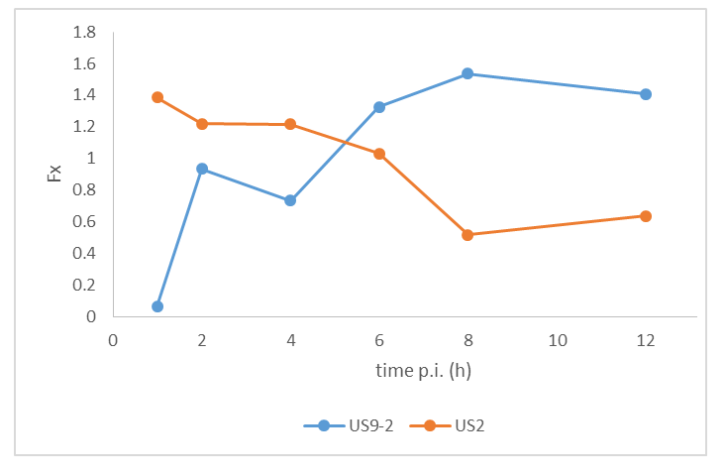


**Supplementary Figure 5.** **5’ cistron variant pairs that presented inverted expression kinetics (Pearson correlation coefficient <-0.5).** The 5’ cistron variants have a common polyA-site, but contain a different number of cistrons.


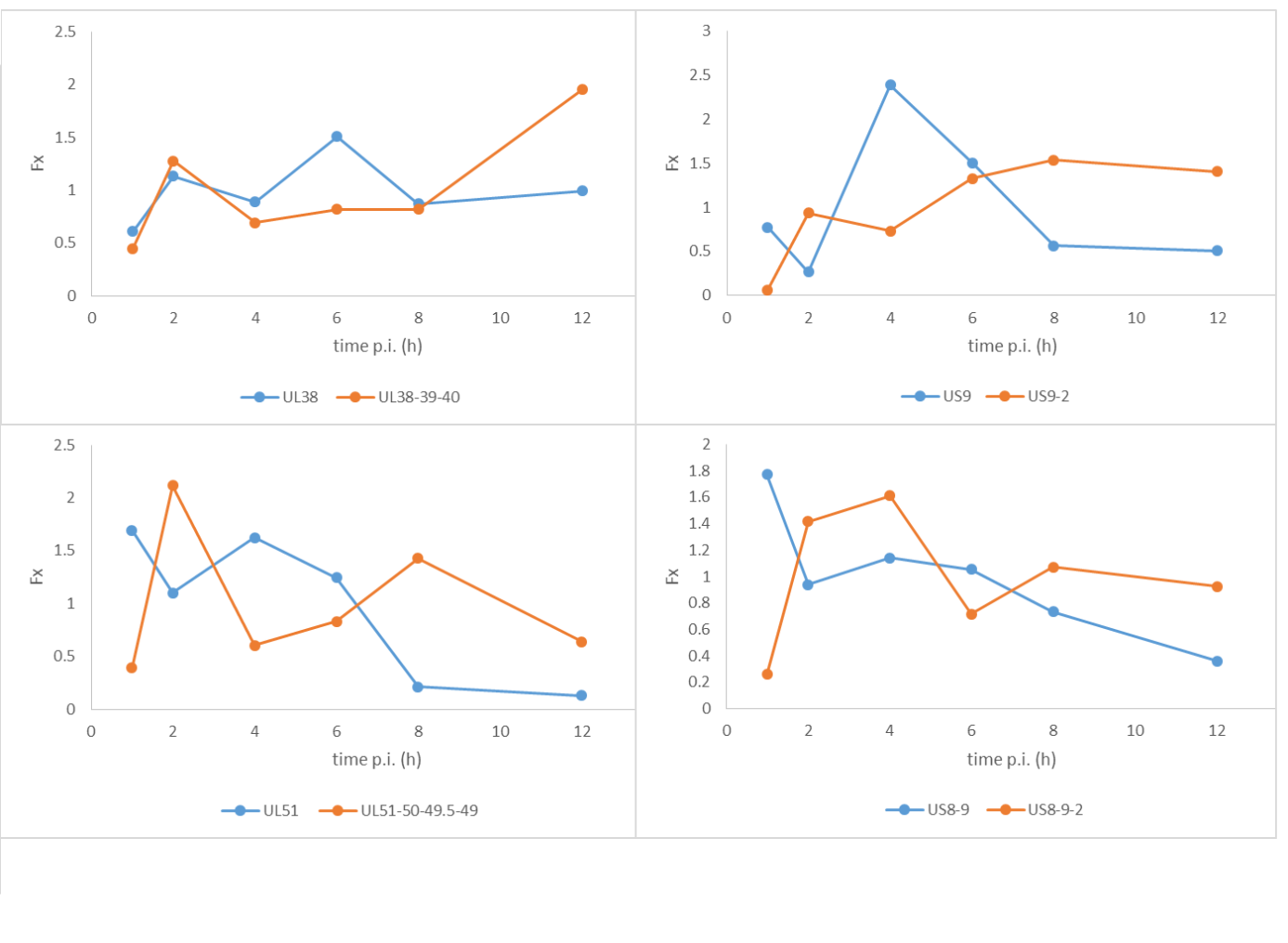


**Supplementary Figure 6. 3’ cistron variant pairs with different expression kinetics (Pearson correlation coefficient ≤0.5, but ≥-0.5).** 3’ cistron variants share a common promoter, but have a different number of cistrons.


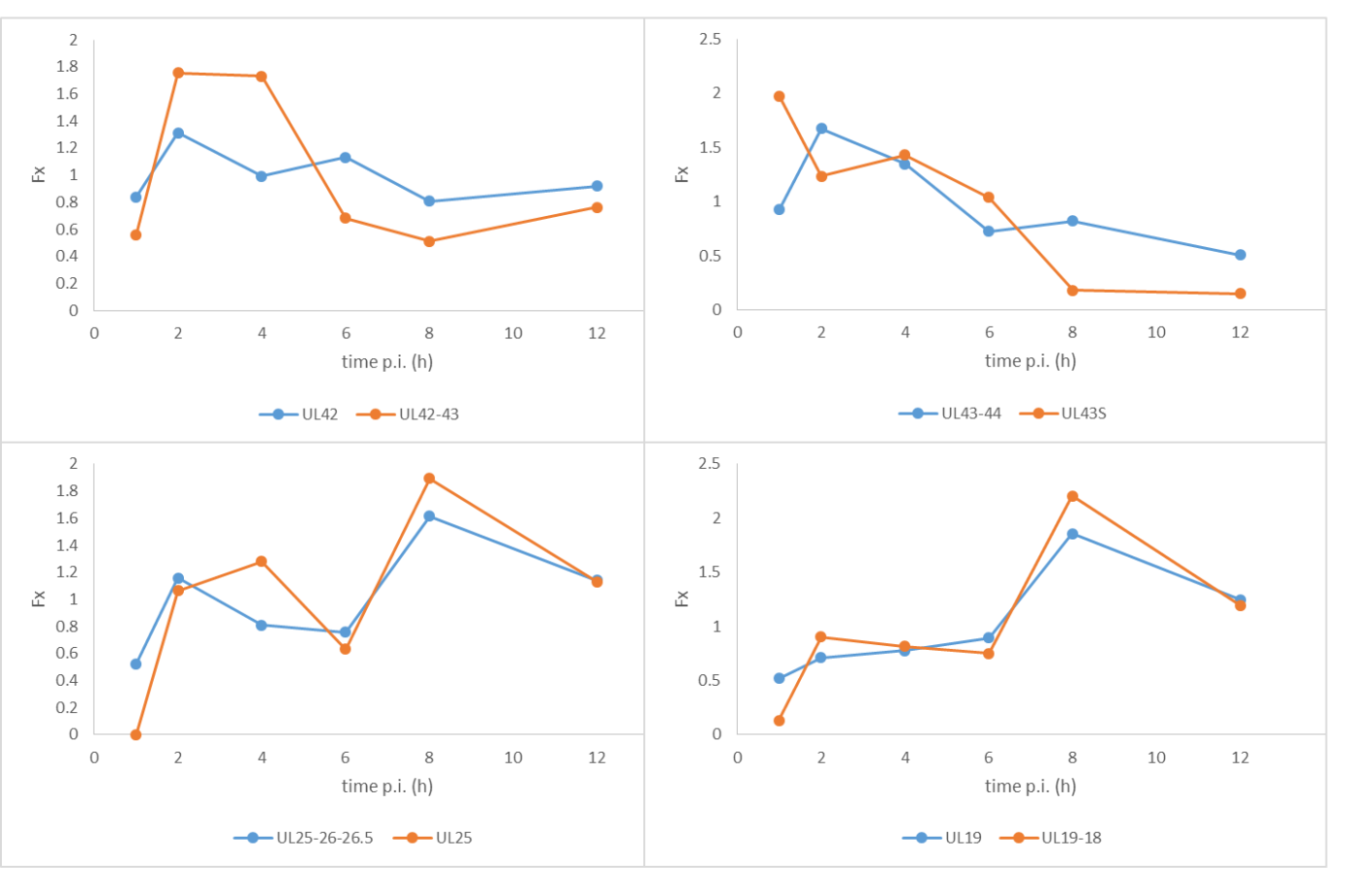


**Supplementary Figure 7. 3’ cistron variant pairs that showed similar expression kinetics (Pearson correlation coefficient >0.5).** 3’ cistron variants share a common promoter, but have a different number of cistrons.


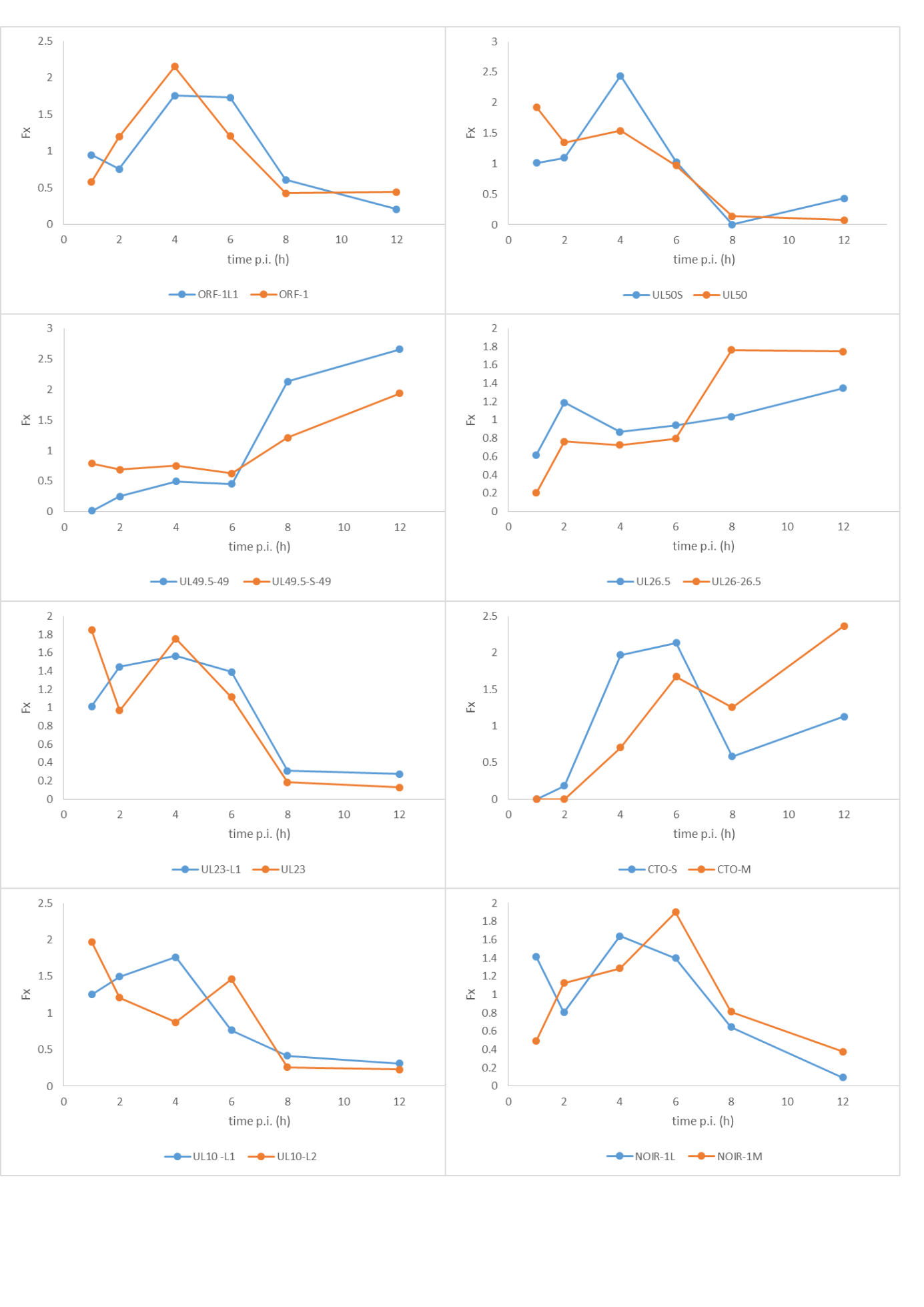

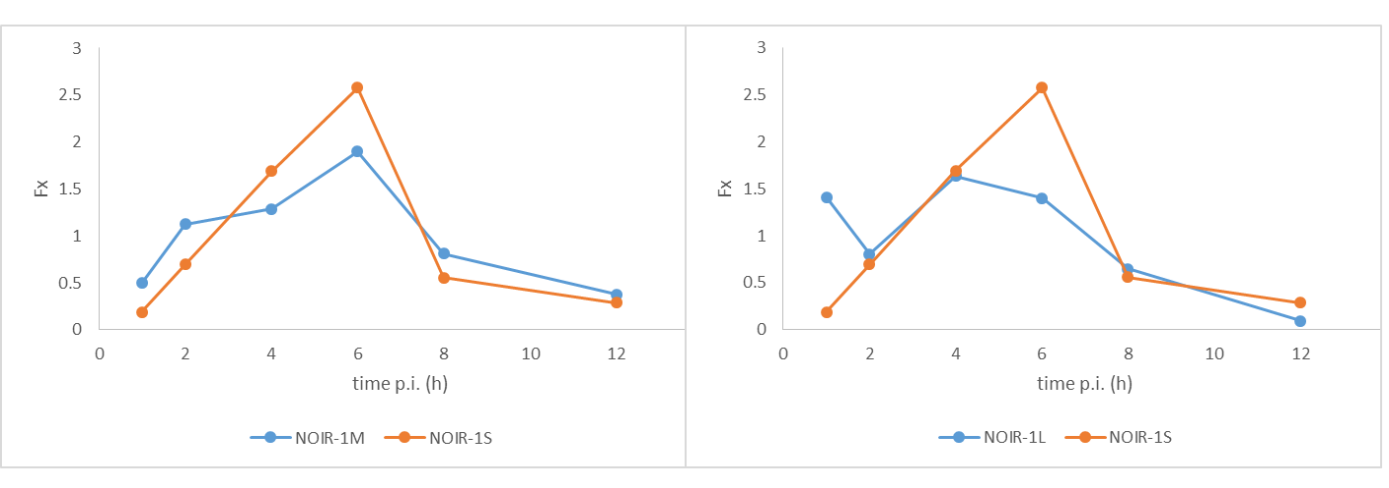


**Supplementary Figure 8. 5’-UTR variant pairs showing similar expression kinetics (Pearson correlation coefficient >0.5).** 5’-UTR variants terminate in the same polyA site, but have different transcriptional start sites.


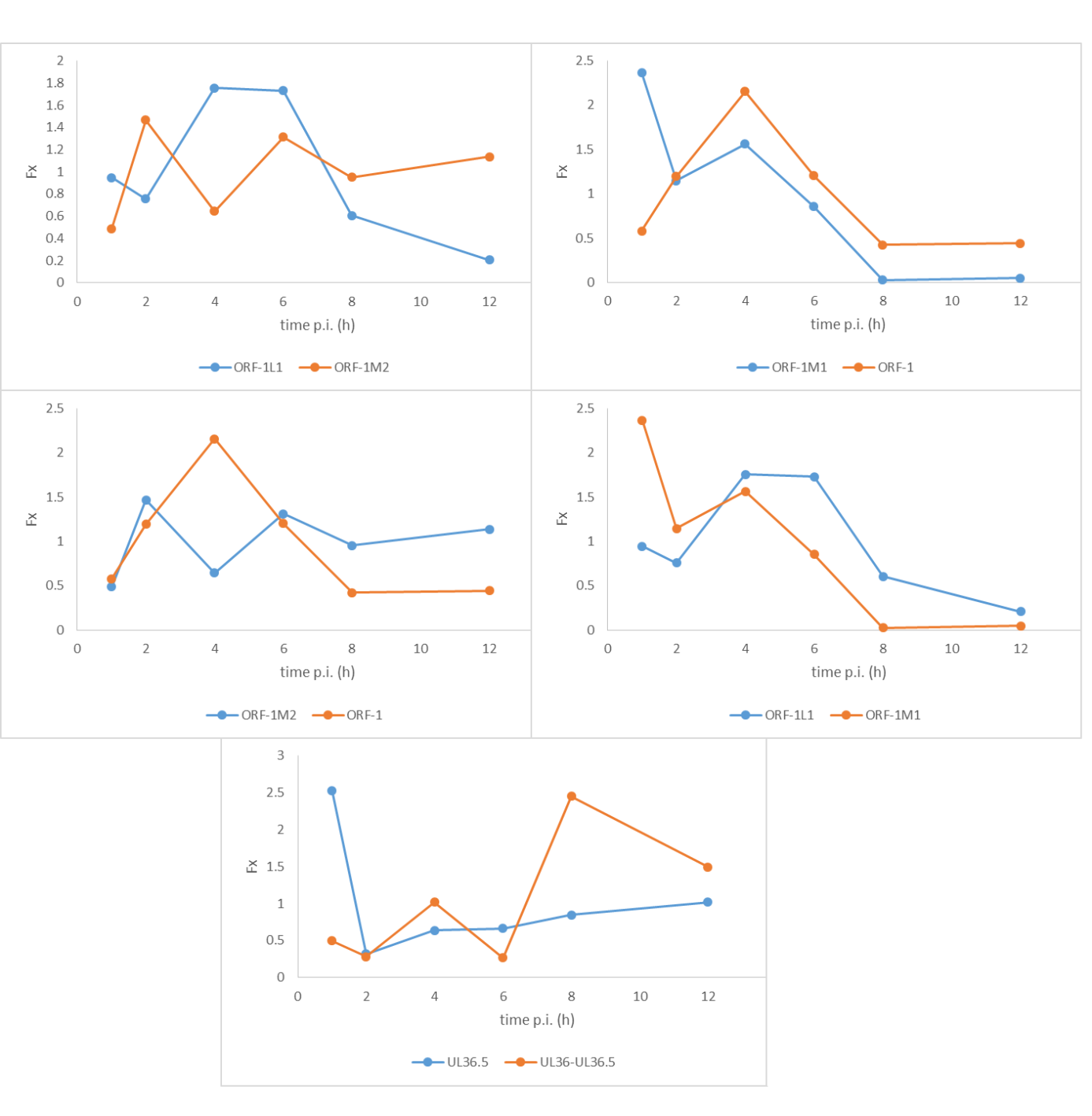


**Figure 9. 5’-UTR variant pairs which showed different expression kinetics (Pearson correlation coefficient ≤0.5, but ≥-0.5).** 5’-UTR variants terminate in the same polyA site, but have different transcriptional start sites.


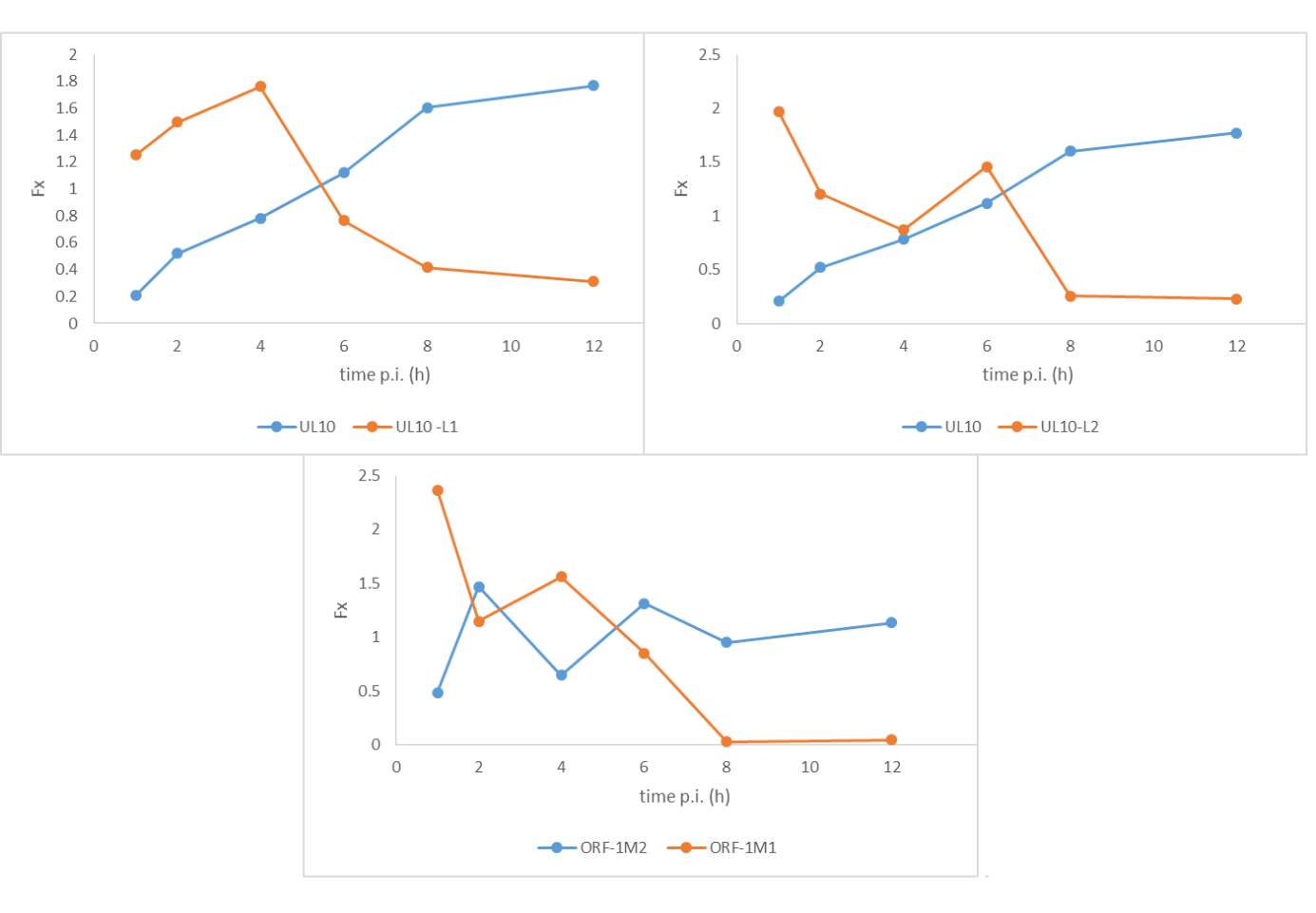


**Supplementary Figure 10. 5’-UTR variant pairs that showed inverted expression kinetics (Pearson correlation coefficient <-0.5).** 5’-UTR variants terminate in the same polyA site, but have different transcriptional start sites.


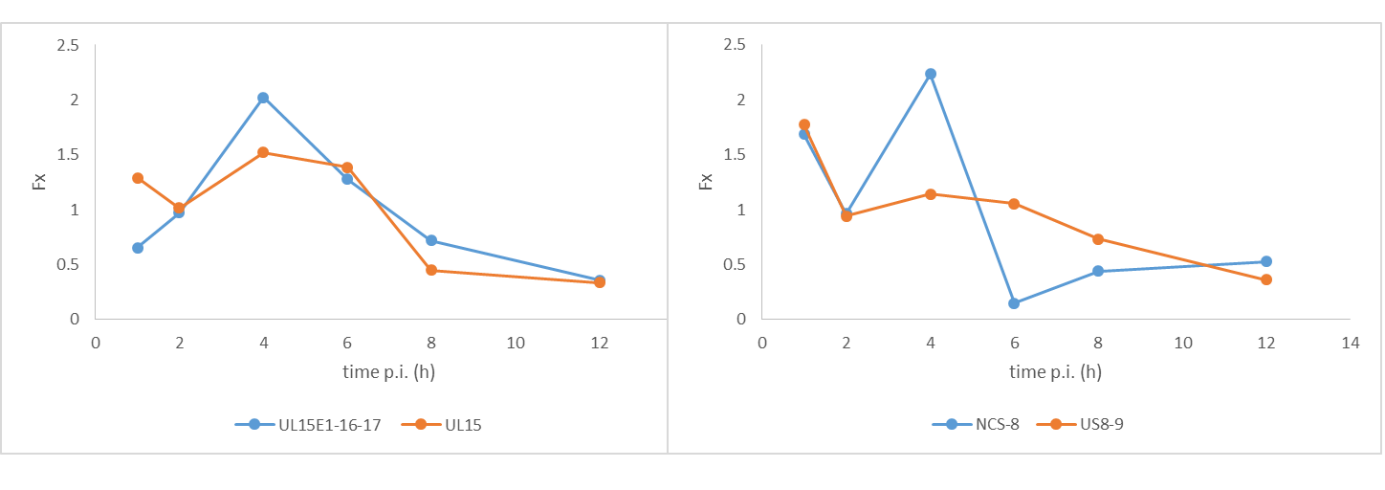


**Supplementary Figure 11. The 3’-UTR variant pairs showed similar expression kinetics (Pearson correlation coefficient >0.5).** 3’-UTR variants share a common promoter, but have different polyA sites.
